# Paricalcitol Attenuates Bleomycin-induced Pulmonary Fibrosis: Insights from Immunohistochemical and Molecular Genetic Analysis

**DOI:** 10.2174/0118715303434362251212040431

**Published:** 2026-04-30

**Authors:** Maxim A. Kriventsov, Darya A. Enzel, Tatyana P. Sataieva

**Affiliations:** 1 Pathological Anatomy Department with Dissection Course, Medical Institute named after S. I. Georgievsky of V. I. Vernadsky CFU, Simferopol, Russia;; 2 Microbiology, Virology and Immunology Department, Medical Institute named after S. I. Georgievsky of V. I. Vernadsky CFU, Simferopol, Russia

**Keywords:** Experimental model, pulmonary fibrosis, paricalcitol, vitamin D receptor, inflammation, remodeling

## Abstract

**Introduction:**

Pulmonary fibrosis is a progressive disease marked by excessive fibrotic transformation of lung tissue. Paricalcitol, a selective vitamin D receptor (VDR) agonist, has shown promise in reducing fibrosis in experimental models, primarily due to its anti-inflammatory and anti-fibrotic properties. This study evaluates the therapeutic effects of paricalcitol on bleomycin-induced pulmonary fibrosis in a rat model.

**Materials and Methods:**

Fifty-four male Wistar rats were randomly assigned to three groups: a control group, a bleomycin-induced pulmonary fibrosis group (PF), and a bleomycin-induced pulmonary fibrosis group with paricalcitol treatment (PF+P). Pulmonary fibrosis was induced *via* intratracheal bleomycin instillation, and paricalcitol was administered every two days to the PF+P group. Effects were assessed through histopathological, immunohistochemical, and molecular genetic analyses, including mRNA expression levels of inflammatory and fibrotic markers at 10, 20, and 30 days.

**Results:**

Paricalcitol treatment significantly reduced fibrosis severity, with the Aschoff score decreasing on day 30 to 2.50±0.63 compared with 5.88±0.52 in untreated PF rats (*p* = 0.02). The number of CD80+ M1 macrophages was reduced on day 10 (29.9±3.8 *vs* 64.2±5.2, *p* = 0.001), while CD163+ M2 macrophages were increased (30.7±2.7 *vs* 21.7±2.8, *p* = 0.03) compared to the control group. Paricalcitol also decreased TGF-β1 expression on day 30 (47.1±5.3 *vs* 70.8±7.1, *p* = 0.03) and MMP-9 on day 20 (28.9±2.5 *vs* 76.4±4.7, *p* < 0.001).

**Discussion:**

The present study investigates the effects of paricalcitol, a selective vitamin D receptor (VDR) agonist, on bleomycin-induced pulmonary fibrosis, revealing notable reductions in inflammatory and fibrotic markers. Our findings indicate that paricalcitol treatment (PF+P group) significantly attenuates fibrosis severity, as observed through histopathological, immunohistochemical, and molecular genetic analyses.

**Conclusion:**

Paricalcitol markedly reduces fibrosis severity, as shown through histopathological, immunohistochemical, and molecular genetic analyses. The treatment decreased the expression of key fibrotic and inflammatory markers, including TGF-β, MMP9, IL-1β, and TNF-α, and shifted macrophage polarization toward an anti-inflammatory M2 phenotype. These findings suggest paricalcitol's potential in modulating the immune response and highlight its therapeutic promise.

## 1 INTRODUCTION

Pulmonary fibrosis is a chronic and debilitating disease characterized by the fibrotic transformation of lung parenchyma, leading to a progressive decline in respiratory function [1, 2]. Despite the availability of several therapies for patients with pulmonary fibrosis, their efficacy remains limited, and a therapeutic gap persists, particularly for agents with immunomodulatory actions [3]. In this context, vitamin D [4, 5], its endogenous metabolites (including CYP11A1-derived derivatives [6]), and a range of synthetic selective vitamin D receptor (VDR) agonists have shown promising positive results in preclinical studies and are plausible disease-modifying candidates targeting key pathogenetic pathways of fibrotic transformation [7, 8].

Paricalcitol, being a synthetic vitamin D analog and selective agonist of VDR, has garnered attention for its anti-inflammatory and anti-fibrotic properties [9, 10]. Initial studies have demonstrated paricalcitol’s potential in modulating immune responses and attenuating fibrotic processes in various organs. For instance, it was shown that paricalcitol can inhibit the proliferation of fibroblasts and reduce the production of extracellular matrix components, which are critical in the pathogenesis of fibrosis [8].

Vitamin D receptor (VDR)-mediated effects play a crucial role in the effects of paricalcitol. The VDR, a nuclear hormone receptor, modulates the expression of numerous genes involved in immune regulation, cellular proliferation, and differentiation [11, 12]. Activation of the VDR by paricalcitol has been shown to exert several protective effects against fibrosis. For instance, VDR activation can inhibit the expression of pro-fibrotic cytokines, such as transforming growth factor-beta (TGF-β), and reduce the activity of fibrotic signaling pathways like TGF-β/Smad and Wnt/β-catenin [8, 13].

In the context of pulmonary fibrosis, VDR-mediated effects are particularly relevant. Pulmonary fibroblasts, the cells primarily responsible for the excessive deposition of extracellular matrix in fibrosis, express VDR, and its activation can mitigate fibroblast proliferation and myofibroblast differentiation [14-16]. Furthermore, VDR activation can suppress inflammation, including systemic inflammatory response, by downregulating the production of pro-inflammatory cytokines such as IL-6 and TNF-α, which are known to contribute to the fibrotic process [17-19].

Experimental studies specifically focusing on pulmonary fibrosis have provided promising data. Animal models treated with paricalcitol have shown reductions in markers of fibrosis, such as collagen deposition and fibroblast activation. These effects are thought to be mediated through the suppression of pro-inflammatory cytokines and the inhibition of key fibrotic signaling pathways [20, 21]. Moreover, VDR activation has been associated with reduced oxidative stress and apoptosis, further contributing to its anti-fibrotic effects [21]. However, despite these encouraging findings, several unresolved issues remain. The precise molecular mechanisms through which VDR-mediated effects may be protective in pulmonary fibrosis are not fully elucidated. Additionally, the long-term efficacy of paricalcitol in chronic models of pulmonary fibrosis requires further investigation [22].

This study aims to evaluate the effects of paricalcitol on bleomycin-induced pulmonary fibrosis, using descriptive histopathological, immunohistochemical, and molecular genetic analyses to provide deeper insights into its therapeutic potential.

## 2 MATERIALS AND METHODS

### 2.1 Animals

Fifty-four male Wistar rats (*Rattus norvegicus*) (8–10 weeks old) weighing 200–220 g were used in the study. All rats were obtained from the certified vivarium of the Medical Institute named after S.I. Georgievsky. The rats had free access to food and water and were randomly housed in standard polycarbonate cages. The controlled environment was maintained at 21–23ºC with 55–60% humidity, and a 12-hour light/dark cycle. The experiment was carried out in April, 2024. The rats were randomly allocated into three experimental groups using a randomization table (Fig. **1**):

1. Control group (CG) (n = 18);
2. Bleomycin-induced pulmonary fibrosis group (PF) (n = 18);
3. Bleomycin-induced pulmonary fibrosis group with paricalcitol treatment (PF+P) (n = 18).

### 2.2 Induction of Pulmonary Fibrosis and Administration of Paricalcitol

After acclimatization of animals, experimental animals (PF and PF+P groups) were anesthetized using an intraperitoneal injection of xylazine (Xyla^®^, Interchemie, Holland) in a dose of 10 mg per kg. The anesthetized rats were placed on a board at a 45-degree angle with their mouths open. A sterile 22-gauge catheter attached to a 1 mL syringe was inserted through the oral cavity into the trachea. Bleomycin sulfate (Veropharm, Russia) (5 mg/kg body weight) dissolved in 0.9% sterile saline was slowly instilled into the trachea. Proper placement of the catheter was ensured by the absence of resistance and confirmation of tracheal location. A control group of rats received an equal volume of 0.9% sterile saline *via* intratracheal instillation following the same procedure as the experimental group.

Animals of the PF+P group (18 rats) were administered paricalcitol (Zemplar^®^, Catalent Pharma Solutions LLC, USA) at a dose of 0.1 mg/kg body weight every 2 days, while rats of the PF group (18 rats) were administered an equivalent volume of 0.9% sterile saline.

The development of pulmonary fibrosis was assessed at various time points post-instillation (days 10, 20, and 30). The rats were euthanized by decapitation under anesthesia, and the lungs were harvested for molecular genetic analysis (mRNA expression), histopathological, and immunohistochemical examination.

### 2.3 Real-time qPCR

Lung tissue was homogenized for RNA isolation, and total RNA was extracted using the RNA-Extran Kit (Syntol^®^, Russia) according to the manufacturer’s protocol. RNA concentration and purity were assessed spectrophotometrically at 260/280 nm with a Nano-500 (Helicon^®^, Russia). Subsequently, 20 ng of RNA was reverse transcribed into cDNA using the MMLV RT kit (Syntol^®^, Russia). Quantitative PCR was performed on a CFX 96 TOUCH Real-Time PCR system (Bio-Rad®, Russia) with a commercial qPCR kit, following the manufacturer’s instructions [23]. Thermal cycling conditions included an initial step at 50°C for 2 minutes, denaturation at 95°C for 10 minutes, and 40 amplification cycles at 95°C for 15 seconds and 60°C for 1 minute. Expression levels of IL-1β, TNF-α, TGF-β, and SMA-α (Table **1**) were quantified, with GAPDH as the reference gene. Relative expression was calculated using the 2−ΔΔCt method [24], where ΔCt represents the difference between target and reference Ct values, and ΔΔCt corresponds to the difference between experimental and control groups. All analyses were conducted by a blinded observer unaware of the group allocations.

### 2.4 Histopathological Analysis

Lung tissue samples were fixed in 10% neutral buffered formalin for approximately 24 hours and then processed using the Logos Hybrid Histological Processor (Milestone Medical^®^, Italy) and embedded with the Leica EG1150 Modular Tissue Embedding Center (Leica Biosystems^®^, Germany). Paraffin blocks were sectioned at ~4 μm thickness with a Leica RM2255 rotary microtome (Leica Biosystems^®^, Germany), and the sections were stained with hematoxylin and eosin (H&E) for microscopic evaluation. Prepared slides were scanned using an Aperio CS2 Digital Pathology Slide Scanner (Leica Biosystems^®^, Germany), and digital image analysis was performed with Aperio ImageScope and ImageJ software [25].

The severity of pulmonary fibrosis was evaluated semi-quantitatively using the modified Aschoff scale from 0 to 8, with higher scores indicating more severe fibrosis [26]:

- Grade 0: No fibrosis
- Grade 1: Minimal fibrous thickening of alveolar or bronchiolar walls
- Grade 2: Moderate thickening of walls without obvious damage to lung architecture
- Grade 3: Increased fibrosis with damage to lung structure and formation of fibrous bands
- Grade 4: Severe distortion of structure and large fibrous areas, with damage extending into adjacent structures
- Grade 5: Total fibrous obliteration of fields
- Grade 6: Severe fibrous obliteration, presence of honeycombing
- Grade 7: Extensive honeycombing and large fibrous masses
- Grade 8: Complete obliteration of the lung architecture with massive fibrosis

### 2.5 Immunohistochemical Analysis

For immunohistochemical analysis, formalin-fixed and paraffin-embedded histological 4 µm sections were used. Staining was performed using a BondMax Semiautomatic Immunohistostainer (Leica Biosystems^®^, Germany). The staining protocol included dewaxing, heat antigen unmasking using Bond Epitope Retrieval 2 solution (Leica Biosystems^®^, Germany) at pH = 9 for 20 minutes at 96°C, blocking peroxidase activity, incubation with the antibody for 15 minutes at room temperature, and visualization using the Bond Polymer Refine Detection system (Leica Biosystems^®^, Germany). For better visualization, counterstaining was performed with hematoxylin according to the standard technique. Histological preparations were scanned using an Aperio CS2 Digital Pathology Slide Scanner (Leica Biosystems^®^, Germany), followed by digital image analysis. The following primary antibodies were used: CD68 (#DF7518; Affinity Biosciences^®^, China); CD80 (#AF5233; Affinity Biosciences^®^, China); CD163 (#PAB726Ra01; Cloud-Clone^®^, China); MMP9 (#PAA553Ra01; Cloud-Clone^®^, China); and TGF-β1 (#PAA124Ra01; Cloud-Clone^®^, China). Quantitative analysis of immunopositive cells was performed on 3 to 5 high-power fields (HPF) per histological slide.

### 2.6 Statistical Analysis

Statistical analysis of the obtained results was conducted using descriptive statistics with Statistica software (Version 10, StatSoft, Inc.). The sample size (n=18 per group) was based on previous studies using similar bleomycin-induced models that achieved statistical power to detect differences in pulmonary fibrosis [8]. The chosen sample size was reviewed and approved by the Institutional Ethics Committee (protocol No. 3 dated 21/03/2023), ensuring compliance with both scientific rigor and animal welfare considerations. Comparative analyses between the control and experimental groups were performed using the Mann-Whitney U Test. Values of *p* < 0.05 were considered to be significant.

### 2.7 Ethics Statements

This study adheres to internationally accepted standards for animal research, following the 3Rs principle. The ARRIVE guidelines were employed for reporting experiments involving live animals, promoting ethical research practices. All procedures were performed under the recommendations of the Guide for the Care and Use of Laboratory Animals [27] and were approved by the local Institutional Ethics Committee (Medical Institute named after S.I. Georgievsky, V.I. Vernadsky Crimean Federal University, Russia; protocol No. 3 dated 21/03/2023). The morphological part of the study and real-time qPCR analysis were carried out in the Central Research Laboratory of the Medical Institute named after S.I. Georgievsky using certified equipment.

## 3 RESULTS

A single intratracheal administration of bleomycin led to a significant loss of body weight in experimental rats compared to the control group, with statistically significant differences persisting through the 30^th^ day of the experiment. Paricalcitol treatment partially mitigated this weight loss, although body weights did not return to control levels (Fig. **2**).

Histopathological evaluation of bleomycin-induced pulmonary fibrosis in rats, following a single intratracheal instillation in the PF group, showed progression from inflammation and early fibrotic changes within the first 10–20 days to established fibrosis by day 30. The early phase (10^th^ day) was characterized by a significant inflammatory response, including diffuse interstitial alveolitis, alveolar destruction, and thickening of the interalveolar septa. In the intermediate phase (20^th^ day), progressive fibrosis was evident, marked by increased fibroblast and myofibroblast proliferation and extracellular matrix deposition. By day 30, fibrotic changes were well established, with extensive collagen deposition and significant disruption of lung parenchyma. As inflammation diminished, lung tissue was increasingly replaced by fibrous tissue foci.

Semi-quantitative assessment using the modified Aschoff scale showed a statistically significant increase in the grade of pulmonary fibrosis from day 10 to day 30 in the PF group (4.66 ± 0.40, 5.03 ± 0.46, and 5.88 ± 0.52 on the 10^th^, 20^th^, and 30^th^ day, respectively) compared to the control group (*p* < 0.001).

Administration of paricalcitol (Zemplar^®^) in the PF+P group reduced the severity of inflammatory and fibrotic changes in the lung parenchyma, as observed in both descriptive histopathological analysis and quantitative assessment using the modified Aschoff scale. However, statistically significant differences between the PF+P and PF groups were observed only on the 30^th^ day of the experiment (2.50 ± 0.63, *p* = 0.02 compared to the PF group).

Typical histopathological changes in the PF and PF+P groups, along with the semi-quantitative assessment of pulmonary fibrosis severity according to the modified Aschoff scale, are shown in Figs. (**3** and **4**).

Following a single intratracheal instillation of bleomycin (PF group), immunohistochemical analysis of the pan-macrophage marker (CD68) revealed a significant presence of macrophages within the mixed lymphohistiocytic infiltrate, with a gradual decrease in CD68+ cells from day 10 to day 30, marking the transition from the acute inflammatory phase to fibrotic transformation. Macrophage polarization data indicated a predominance of the pro-inflammatory M1 macrophage subpopulation (CD80+) in the early stages of the experiment (day 10 – 64.2 ± 5.2 per HPF), with a gradual decrease in their numbers on days 20 and 30 (44.4 ± 5.4 per HPF and 27.8 ± 2.9 per HPF, respectively). Conversely, the M2 macrophage subpopulation increased progressively in the later stages (20^th^ and 30^th^ days – 30.9 ± 2.8 per HPF and 35.9 ± 2.9 per HPF, respectively).

These findings on macrophage subpopulation distribution align with the immunohistochemical data on extracellular matrix remodeling and fibrotic transformation markers (MMP9, TGF-β1), showing increasing TGF-β1 expression from day 10 to day 30 as fibrosis progressed, alongside an initial increase in MMP9 expression in the early stages, which gradually decreased by day 30.

In the PF+P group, paricalcitol treatment resulted in a general reduction in the number of CD68+ cells across all time points (most notably on day 10 – 49.8 ± 6.5 per HPF, *p* = 0.003), along with a significant reduction in the M1 macrophage subpopulation on day 10 (29.9 ± 3.8 per HPF, *p* = 0.001). The M2 macrophage subpopulation (CD163+) showed a significant increase in the early stage (day 10 – 30.7 ± 2.7 per HPF, *p* = 0.03), followed by a statistically significant decrease in later stages compared to the PF group (20^th^ and 30^th^ days – 20.7 ± 2.3 per HPF, *p* = 0.02 and 23.6 ± 2.6 per HPF, *p* = 0.01, respectively). Additionally, TGF-β1 expression significantly decreased on days 10 and 30 (22.2 ± 2.9 per HPF, *p* = 0.01 and 47.1 ± 5.3 per HPF, *p* = 0.03), as did MMP9 expression on days 10 and 20 (59.2 ± 3.2 per HPF, *p* = 0.001 and 28.9 ± 2.5 per HPF, *p* < 0.001).

Typical patterns of immunohistochemical expression of macrophage markers (CD68, CD80, and CD163) and fibrotic transformation markers (MMP9 and TGF-β1) in the PF and PF+P groups are shown in Figs.(**5** and **6**). Results from the quantitative evaluation of immunopositive cells in the PF and PF+P groups are presented in Table **2**.

The mRNA expression levels of IL-1β, TNF-α, TGF-β, and SMA-α in lung tissue homogenates were quantified and compared between the PF and PF+P groups at 10-, 20-, and 30-days post-bleomycin instillation. The results were consistent with the findings from the histological and immunohistochemical analyses. Specifically, in the PF group, IL-1β mRNA expression was significantly elevated on day 10 (*p* < 0.001), indicating a peak in the acute inflammatory response. IL-1β levels decreased by days 20 and 30 but remained above baseline. In contrast, the PF+P group showed a marked reduction in IL-1β expression at all time points compared to the PF group, with a statistically significant difference observed on day 30 (*p* = 0.03).

TNF-α expression followed a similar pattern to IL-1β, with initial elevation and subsequent reduction over time. Conversely, TGF-β mRNA expression, which is associated with fibrotic activity, progressively increased in the PF group from day 10 to day 30 (*p* < 0.001 at all terms of the experiment), correlating with the transition from inflammation to established fibrosis. In the PF+P group, TGF-β levels were significantly lower than in the PF group on both days 20 and 30 (*p* = 0.03 and *p* = 0.01 on day 20 and day 30, respectively), indicating that paricalcitol treatment reduced the activation of profibrotic signaling pathways.

The myofibroblast activation marker, SMA-α, was progressively upregulated in the PF group, reaching its highest expression on day 30 (*p* < 0.001 compared to the control group), consistent with extensive fibrosis development. In the PF+P group, SMA-α expression was significantly reduced on days 20 and 30 compared to the PF group (both *p* = 0.04 on day 20 and day 30), demonstrating that paricalcitol mitigated myofibroblast differentiation and fibrotic progression.

The fold-changes in relative mRNA expression in the control, PF, and PF+P groups are presented in Fig. (**7**).

## 4 DISCUSSION

The present study investigates the effects of paricalcitol, a selective vitamin D receptor (VDR) agonist, on bleomycin-induced pulmonary fibrosis, revealing notable reductions in inflammatory and fibrotic markers. Our findings indicate that paricalcitol treatment (PF+P group) significantly attenuates fibrosis severity, as observed through histopathological, immunohistochemical, and molecular genetic analyses.

Paricalcitol treatment significantly reduced fibrosis severity by the end of the study period. In the PF group, fibrosis progression was evident, with extensive collagen deposition, thickening of interalveolar septa, and destruction of lung parenchyma by day 30. However, paricalcitol administration led to a statistically significant reduction in fibrosis scores on the Aschoff scale compared to the untreated PF group, particularly on day 30 of the experiment.

Immunohistochemical analysis demonstrated the effect of paricalcitol on shifting macrophage populations (M1 and M2 macrophages). In untreated PF animals, there was a predominance of CD80+ M1 macrophages in the early stages, transitioning to increased CD163+ M2 macrophages by day 30, corresponding to the natural progression from inflammation to tissue remodeling. Paricalcitol treatment enhanced this transition by reducing M1 macrophages and increasing the M2 macrophage subpopulation. This early shift suggests that paricalcitol may help mitigate prolonged inflammation and promote the reparative phase, consistent with findings that VDR activation facilitates anti-inflammatory macrophage polarization [28, 29].

Paricalcitol administration also led to a reduction in key fibrotic markers, specifically TGF-β1 and MMP9. TGF-β1, a pivotal cytokine mediating fibroblast differentiation and extracellular matrix (ECM) deposition, was notably lower in the PF+P group compared to the PF group at days 10 and 30. Similarly, MMP9 levels, which were elevated during early fibrosis stages, were reduced by paricalcitol treatment, indicating decreased ECM remodeling activity. These results support the hypothesis that paricalcitol can modulate ECM dynamics and suppress pathways leading to excessive collagen deposition [30, 31].

The PF+P group also exhibited significantly lower mRNA expression levels of inflammatory (IL-1β, TNF-α) and pro-fibrotic (TGF-β, SMA-α) markers compared to the PF group at days 20 and 30. This reduction in both inflammation and fibrosis markers suggests that paricalcitol administration effectively attenuates the progression of bleomycin-induced lung fibrosis in this model.

The beneficial effects of paricalcitol observed in this study are likely mediated through multiple pathways involving immune modulation and inhibition of fibrotic signaling. Based on recent evidence, the antifibrotic activity of VDR activation is attributed to its multilevel regulation of key profibrotic and pro-inflammatory signaling cascades. Ligand-associated activation of VDR can interfere with central pathways involved in fibrotic remodeling, including SMAD [32, 33], p38 MAPK [34], NF-κB [35], JAK/STAT [36], PPAR-α/γ [37, 38], and calcineurin/NFAT signaling [39, 40]. In addition, VDR may exert transcriptional control over several cytoprotective signaling systems such as Nrf2 [41], thereby contributing to antioxidant defense. A particularly relevant mechanism in lung fibrosis involves VDR-dependent suppression of the renin-angiotensin system through inhibition of renin production, accompanied by downregulation of TGF-β/SMAD and p38 MAPK pathways [42]. Since renin-angiotensin mediators—especially angiotensin II—intensify extracellular matrix deposition and enhance TGF-β expression during fibrotic progression [16], inhibition of this axis represents a key antifibrotic effect of VDR signaling. Overall, the vitamin D/VDR complex functions as a transcription factor that modulates cellular proliferation and differentiation and contributes to immune regulation, thereby counteracting both inflammatory and structural drivers of pulmonary fibrosis.

VDR activation by paricalcitol reduced the expression of TGF-β and MMP9, which act as orchestrators of fibroblast activation and extracellular matrix deposition [43, 44]. Additionally, paricalcitol’s impact on inflammatory markers such as IL-1β and TNF-α suggests that it can reduce the chronic inflammation that typically precedes fibrosis [28]. The suppression of TGF-β/Smad and Wnt/β-catenin pathways observed in the present study, as well as in other experimental studies, supports the hypothesis that paricalcitol’s antifibrotic effects are related to its modulation of VDR-dependent mechanisms [8]. Moreover, macrophage subpopulations are key cellular elements regulating the transition from pro-inflammatory to anti-inflammatory responses in tissue. Macrophage polarization plays a critical role in the progression of pulmonary fibrosis, particularly through M1 (CD80+) and M2 (CD163+) subtypes, which drive inflammation and tissue repair, respectively [45-47]. In the PF group, an initial predominance of pro-inflammatory M1 macrophages gradually shifted to M2 macrophages as fibrosis progressed, consistent with the typical immune response observed in fibrotic lung injury [48, 49]. Importantly, paricalcitol treatment led to an earlier shift toward M2 macrophage polarization, suggesting that VDR activation may enhance the anti-inflammatory, reparative phase of the immune response. This aligns with previous findings showing that vitamin D and its analogs can modulate macrophage activity and polarization in fibrotic and inflammatory processes [28, 50].

Previous studies have investigated paricalcitol’s antifibrotic properties, although data specifically related to pulmonary fibrosis remain limited. In renal fibrosis models, paricalcitol reduced collagen deposition and fibroblast activation through downregulation of TGF-β and inhibition of fibrotic signaling pathways [9, 10]. While other vitamin D analogs, such as calcitriol, have shown benefits in pulmonary models, the effects of paricalcitol in pulmonary fibrosis have been less explored [14]. The results of the present study are consistent with findings in cardiac, kidney, and liver fibrosis models, demonstrating that paricalcitol can downregulate profibrotic markers [11, 51].

Experimental rodent models of pulmonary fibrosis, though limited, primarily employ methods such as intratracheal instillation [52, 53] or aerosolized bleomycin inhalation [54, 55]. These models have substantially advanced understanding of the cellular and molecular mechanisms of pulmonary fibrosis and highlighted the potential therapeutic value of vitamin D and its analogs. For instance, in a rodent model of bleomycin-induced fibrosis, vitamin D administration markedly reduced fibrotic indicators, including hydroxyproline content, smooth muscle actin, and TGF-β expression, as well as histopathological and ultrastructural lung alterations [56].

In bleomycin-induced fibrosis, vitamin D also reduced mRNA expression levels of collagen types I and III, as well as smooth muscle actin, while restoring VDR mRNA levels that were initially reduced by bleomycin exposure. One proposed mechanism for these anti-fibrotic effects involves the suppression of TGF-β1-induced SMAD phosphorylation [57]. Another study using a C57BL/6 mouse model of bleomycin-induced fibrosis demonstrated that treatment with the vitamin D receptor agonist paricalcitol significantly prevented body weight loss and mitigated fibrotic changes in the lung. In contrast, vitamin D deficiency led to more severe lung damage. Paricalcitol treatment decreased the expression of fibrosis-related markers, including TGF-β, α-SMA, type I collagen, and fibronectin, as well as components of the renin-angiotensin system, such as angiotensinogen, angiotensin II, and type I angiotensin receptors [42].

While generally consistent with the results of previous experimental studies, the present study provides novel insights specific to experimental pulmonary fibrosis and VDR-mediated effects. The results demonstrate an early shift in macrophage polarization within the lung parenchyma, suggesting that paricalcitol’s effects on M1 and M2 macrophages may represent a key mechanism for modulating the inflammatory phase of pulmonary fibrosis. These observations, based on histopathological and immunohistochemical analyses, are further supported by detailed analysis of mRNA expression in lung tissue, providing molecular genetic evidence for paricalcitol’s impact on fibrotic and inflammatory pathways.

Although the present experimental model does not fully replicate the complexity and chronic progression of human pulmonary fibrosis, the findings suggest several mechanistic pathways with potential translational relevance. Vitamin D deficiency is common among patients with chronic lung diseases, including idiopathic pulmonary fibrosis, and is associated with disease severity and dysregulated immune responses [58, 59]. Selective activation of VDR, as achieved by paricalcitol, may help inhibit persistent pro-fibrotic signaling driven by TGF-β and excessive extracellular matrix deposition observed in idiopathic pulmonary fibrosis [60]. The demonstrated effects of paricalcitol, including attenuation of fibrosis severity (Aschoff score reduction to 2.50 ± 0.63 *vs* 5.88 ± 0.52 in the untreated group on Day 30), suppression of TGF-β1 and MMP-9 expression, and promotion of M2 macrophage polarization, highlight its potential therapeutic benefits in limiting progressive tissue remodeling and enhancing endogenous resolution mechanisms.

While current antifibrotic therapies used in clinical practice, such as pirfenidone and nintedanib [61-63], may slow but do not reverse structural damage completely, VDR-targeted approaches may offer complementary mechanisms, particularly in modifying the early inflammatory cascade. The safety profile of paricalcitol, which has been widely used in nephrology patients [64, 65], further supports its use in clinical settings in fibrotic lung disease.

The results of this study encourage further investigation of VDR-mediated effects in pulmonary fibrosis. Further researches are needed to study the molecular pathways mediated by paricalcitol, specifically from the point of view of its long-term efficiency and safety in pulmonary fibrosis. Understanding the mechanisms of the ligand-associated activation of VDR receptors can open up further perspectives for translating these findings into clinical settings.

## CONCLUSION

The present study provides evidence that paricalcitol, a selective vitamin D receptor (VDR) agonist, exerts significant protective effects in a bleomycin-induced model of pulmonary fibrosis. Paricalcitol treatment led to a marked reduction in fibrosis severity, as demonstrated by histopathological, immunohistochemical, and molecular analyses, which showed decreased expression of key fibrotic and inflammatory markers, including TGF-β, MMP9, IL-1β, and TNF-α. Additionally, paricalcitol promoted an early shift in macrophage polarization toward the anti-inflammatory M2 phenotype, highlighting its potential role in modulating the immune response.

These findings align with existing studies on vitamin D analogs, suggesting that paricalcitol may offer a promising therapeutic approach by inhibiting pro-fibrotic signaling pathways, reducing extracellular matrix deposition, and attenuating inflammatory responses in the lung parenchyma. Overall, this research enhances our understanding of VDR agonists in pulmonary fibrosis and supports the potential use of paricalcitol as a novel therapy for this condition.

## STUDY LIMITATIONS

While the present study demonstrates significant protective effects of paricalcitol in a bleomycin-induced model of pulmonary fibrosis, several limitations should be acknowledged. First, the study employed a single experimental model of pulmonary fibrosis. Although the bleomycin model is widely used and well established, it does not fully recapitulate the complex, progressive, and heterogeneous nature of human pulmonary fibrosis. Second, the study focused on selected inflammatory and fibrotic markers (IL-1β, TNF-α, TGF-β, SMA-α, MMP9) and macrophage polarization but did not assess broader signaling pathways (*e.g.*, Wnt/β-catenin, NF-κB, or renin–angiotensin system pathways), which could provide further insight into the mechanistic effects of paricalcitol. Finally, translation of findings from rodents to humans should be approached with caution, as pharmacokinetics, immune responses, and fibrotic remodeling may differ across species.

## Figures and Tables

**Fig. (1):**
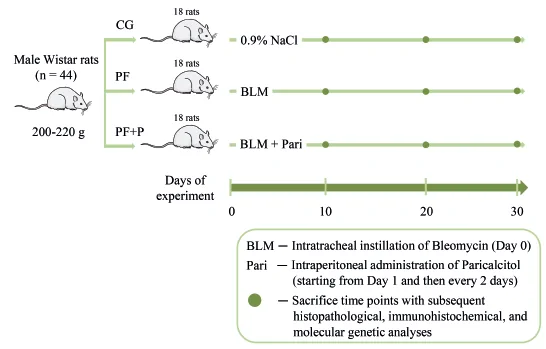
Scheme of the experiment.

**Fig. (2):**
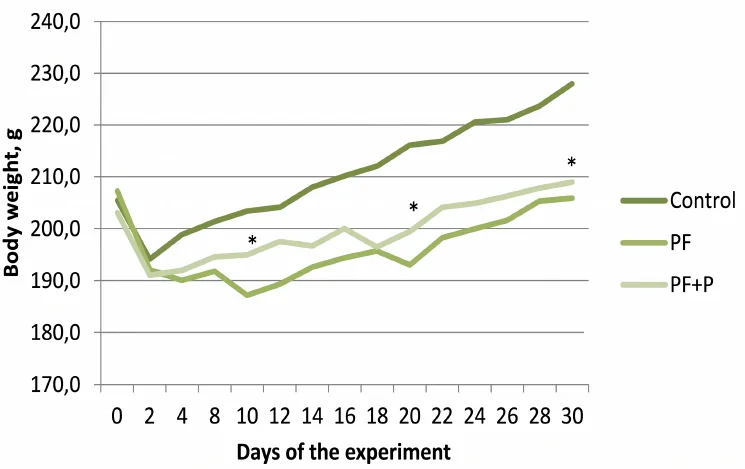
Dynamics of the changes of body weight of the rats in control, PF, and PF+P groups during the experiment.

**Fig. (3):**
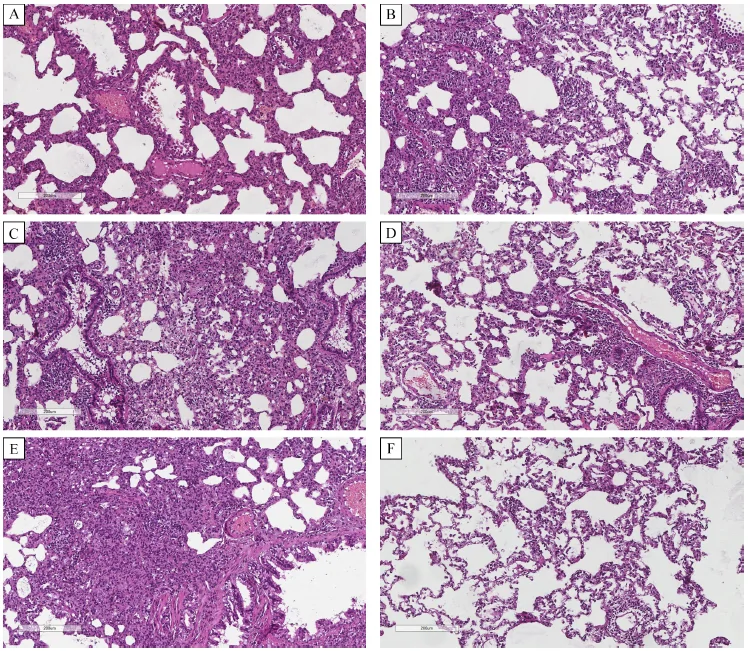
Histopathological evaluation of the lung parenchyma after single intratracheal instillation of bleomycin. (**A**) – PF group, 10 days. Thickening of the interalveolar septa (arrows) along with diffuse inflammatory changes and microvasculature thrombosis (arrowheads), x400; (**B**) – PF+P group 10 days. Thickening of the interalveolar septa and alveolitis (arrows) with some preserved alveoli (arrowheads), x200; (**C**) – PF group, 20 days. Formation of the fibrous foci (arrows) with peribronchiolar lymphocytic infiltration (arrowheads), x200; (**D**) – PF+P group 20 days. Foci of fibrous perivascular and peribronchial transformation of the pulmonary tissue (arrows), x200. (**E**) – PF group, 30 days. Complete fibrous obliteration (arrows) with mature peribronchial fibrosis (arrowheads) and honeycomb-like structures, x200; (**F**) – PF+P group 30 days. Minimal to moderate thickening of the interalveolar septa with mild inflammatory changes (arrows), x200. H&E stain.

**Fig. (4):**
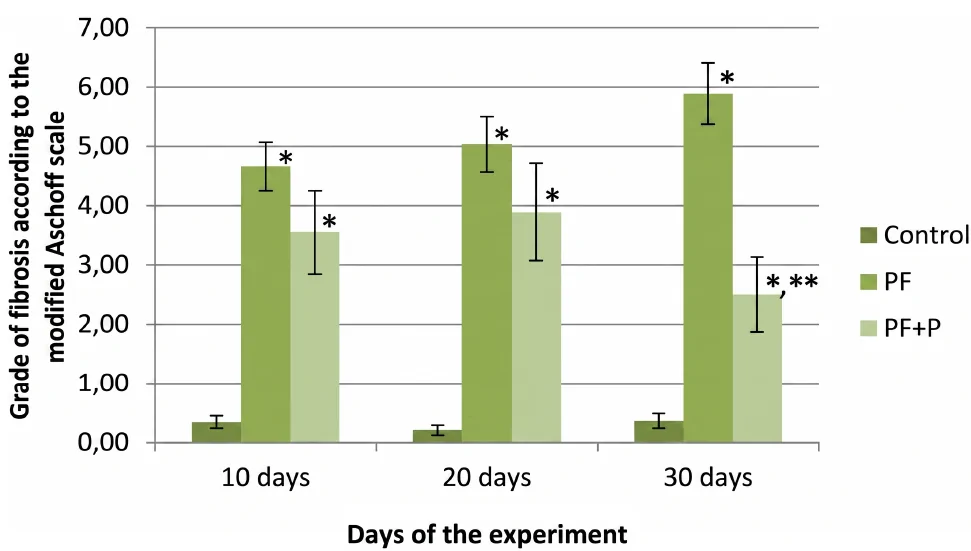
Results of the quantitative assessment of the pulmonary fibrosis using the modified Aschoff scale in control, PF, and PF+P groups. * – statistically significant differences compared to the control group; ** – statistically significant differences compared to the PF group.

**Fig. (5):**
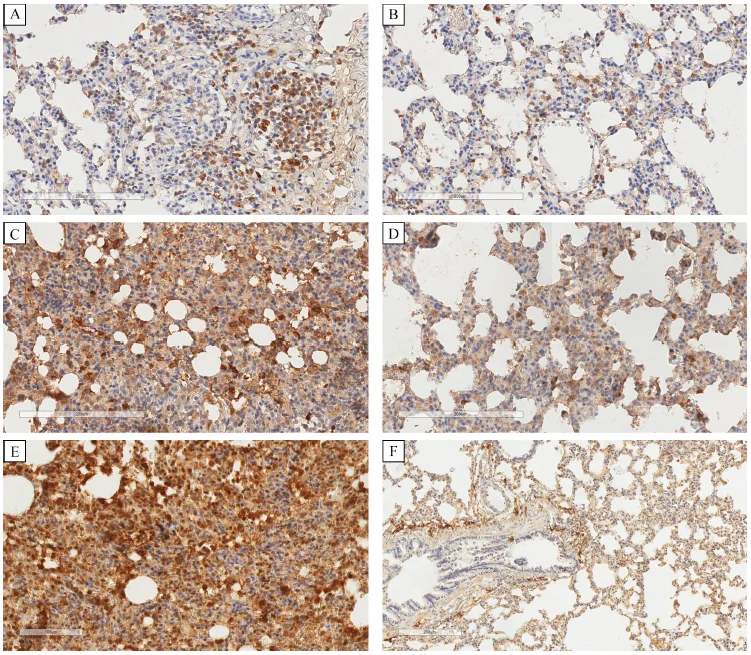
Patterns of the immunohistochemical expression of macrophage markers (CD68, CD80, and CD163) in PF and PF+P groups. (**A**) – CD68, PF group, 10 days; focal accumulation of CD68+ cells within mixed inflammatory infiltrate (arrows), x600; (**B**) – CD68; PF+P group, 10 days; diffuse distribution of CD68+ cells (arrows) within thickened alveolar septa, x600; (**C**) – CD80, PF group, 20 days; diffuse accumulation of proinflammatory CD80+ cells (arrows) in the area of the fibrous transformation, x600; (**D**) – CD80, PF+P group, 20 days; less number of CD80+ cells (arrows) in lung parenchyma compared to untreated group, x600; (**E**) – CD163, PF group, 20 days; presence of massive focal accumulation of CD163+ cells (arrows) in fibrotic airless area, x600; (**F**) – CD163, PF+P group, 20 days; local (predominantly, peribronchial) accumulation of CD163+ cells (arrows), x400.

**Fig. (6):**
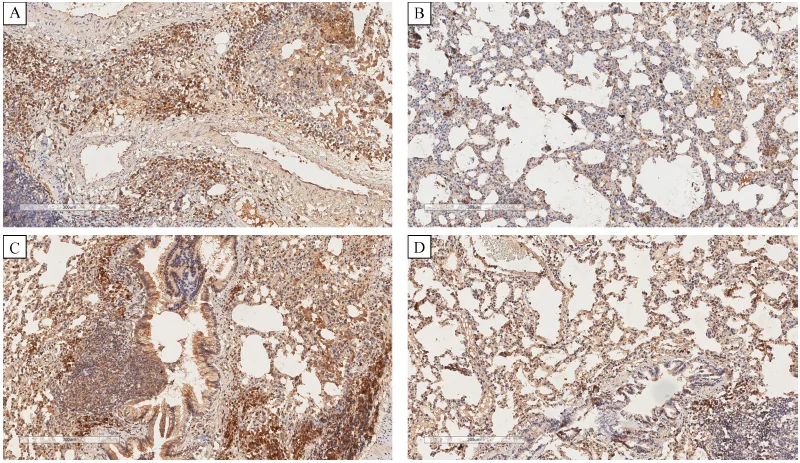
– Immunohistochemical expression of MMP-9 and TGF-β1 in PF and PF+P groups. (**A**) – MMP-9, PF group, 20 days; pronounced immunohistochemical expression of MMP-9 (arrows) in cells of the mixed inflammatory infiltrate, x200; (**B**) – MMP-9, PF+P group, 20 days; single diffuse immunopositive MMP-9+ cells (arrows) in lung parenchyma, x400; (**C**) – TGF-β1, PF group, 30 days; massive accumulation of TGF-β1 immunopositive cells (arrows) within peribronchial fibrotic area, x200; (**D**) – TGF-β1, PF+P group, 30 days; presence of single focal accumulations of immunopositive TGF-β1+ cells (arrows), x400.

**Fig. (7):**
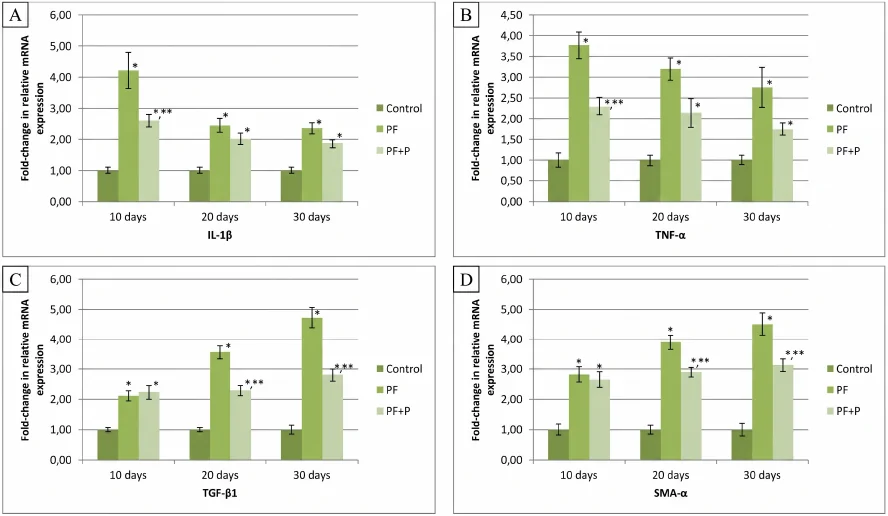
– The bars represent geometric mean fold changes in relative mRNA expression of IL-1β (**A**), TNF-α (**B**), TGF-β1 (**C**), and SMA-α (**D**), and the vertical lines represent the standard errors of the mean fold change in each experimental group. mRNA expression was normalized to the expression of the housekeeping GAPDH gene. Notes: * – statistically significant differences compared to the control group; ** – statistically significant differences compared to the PF group.

**Table 1:** Primers used for RT-qPCR.

**mRNA**	**Forward (F) Primer Sequence**	**Reverse (R) Primer Sequence**
GAPDH	TGCCACTCAGAAGACTGTGG	TTCAGCTCTGGGATGACCTT
IL-1β	TTGAGTCTGCACAGTTCCCC	GTCCTGGGGAAGGCATTAGG
TNF-α	CCAGGTTCTCTTCAAGGGACAA	CTCCTGGTATGAAATGGCAAATC
TGF-β	GACCGCAACAACGCAATCTA	GACAGCAATGGGGGTTCTGG
SMA-α	CCTCTTCCAGCCATCTTTCAT	CGAGAGGACGTTGTTAGCATAG

**Table 2:** Results of the quantitative evaluation of the number of immunopositive cells in PF and PF+P groups.

-	10 days		20 days		30 days	
Markers	PF	PF+P	PF	PF+P	PF	PF+P
CD68	81.6±3.9	49.8±6.5* (*p*=0.003)	58.9±4.9	43.6±6.4* (*p*=0.03)	55.5±6.3	34.7±3.0* (*p*=0.03)
CD80	64.2±5.2	29.9±3.8* (*p*=0.001)	44.4±5.4	29.6±1.7* (*p*=0.04)	27.8±2.9	24.3±2.2 (*p*=0.38)
CD163	21.7±2.8	30.7±2.7* (*p*=0.03)	30.9±2.8	20.7±2.3* (*p*=0.02)	35.9±2.9	23.6±2.6* (*p*=0.01)
MMP-9	89.4±4.2	59.2±3.2* (*p*=0.001)	76.4±4.7	28.9±2.5* (*p*<0.001)	39.3±4.6	33.6±4.8 (*p*=0.33)
TGF-β1	34.1±3.6	22.2±2.9* (*p*=0.01)	62.0±8.6	43.1±4.5 (*p*=0.19)	70.8±7.1	47.1±5.3* (*p*=0.03)

**Note:** * statistically significant differences compared to the PF group.

## Data Availability

The datasets generated and/or analyzed during the current study are available from the corresponding author on reasonable request.

## LIST OF ABBREVIATIONS

CD68: Cluster of Differentiation 68
CD80: Cluster of Differentiation 80
CD163: Cluster of Differentiation 163
CG: Control Group
ECM: Extracellular Matrix
GAPDH: Glyceraldehyde-3-Phosphate Dehydrogenase
H&E: Hematoxylin and Eosin
HPF: High-Power Field
IL-1β: Interleukin-1 beta
IL-6: Interleukin-6
MAPK: Mitogen-Activated Protein Kinase
MMP9: Matrix Metalloproteinase 9
mRNA: Messenger Ribonucleic Acid
NF-κB: Nuclear Factor kappa-light-chain-enhancer of activated B cells
PF: Pulmonary Fibrosis
PF+P: Pulmonary Fibrosis with Paricalcitol Treatment
qPCR: Quantitative Polymerase Chain Reaction
RT-qPCR: Reverse Transcription Quantitative Polymerase Chain Reaction
SMA-α: Alpha Smooth Muscle Actin
TGF-β: Transforming Growth Factor Beta
TNF-α: Tumor Necrosis Factor Alpha
VDR: Vitamin D Receptor
